# Semantic segmentation to identify bladder layers from H&E Images

**DOI:** 10.1186/s13000-020-01002-1

**Published:** 2020-07-16

**Authors:** Muhammad Khalid Khan Niazi, Enes Yazgan, Thomas E. Tavolara, Wencheng Li, Cheryl T. Lee, Anil Parwani, Metin N. Gurcan

**Affiliations:** 1 Center for Biomedical Informatics, Wake Forest School of Medicine, Winston-Salem, NC USA; 2 Department of Pathology, Wake Forest School of Medicine, Winston-Salem, NC USA; 3 Department of Urology, The Ohio State University, Columbus, OH USA; 4 Department of Pathology, The Ohio State University, Columbus, OH USA

## Abstract

**Background:**

Identification of bladder layers is a necessary prerequisite to bladder cancer diagnosis and prognosis. We present a method of multi-class image segmentation, which recognizes urothelium, lamina propria, muscularis propria, and muscularis mucosa layers as well as regions of red blood cells, cauterized tissue, and inflamed tissue from images of hematoxylin and eosin stained slides of bladder biopsies.

**Methods:**

Segmentation is carried out using a U-Net architecture. The number of layers was either, eight, ten, or twelve and combined with a weight initializers of He uniform, He normal, Glorot uniform, and Glorot normal. The most optimal of these parameters was found by through a seven-fold training, validation, and testing of a dataset of 39 whole slide images of T1 bladder biopsies.

**Results:**

The most optimal model was a twelve layer U-net using He normal initializer. Initial visual evaluation by an experienced pathologist on an independent set of 15 slides segmented by our method yielded an average score of 8.93 ± 0.6 out of 10 for segmentation accuracy. It took only 23 min for the pathologist to review 15 slides (1.53 min/slide) with the computer annotations. To assess the generalizability of the proposed model, we acquired an additional independent set of 53 whole slide images and segmented them using our method. Visual examination by a different experienced pathologist yielded an average score of 8.87 ± 0.63 out of 10 for segmentation accuracy.

**Conclusions:**

Our preliminary findings suggest that predictions of our model can minimize the time needed by pathologists to annotate slides. Moreover, the method has the potential to identify the bladder layers accurately. Further development can assist the pathologist with the diagnosis of T1 bladder cancer.

## Introduction

Bladder cancer remains a prevalent disease in the US. In 2020, an estimated 62,100 men and 19,300 women will be diagnosed with the disease, and another 17,670 individuals are expected to die from it [1]. In particular, the treatment of high-grade T1 bladder cancer, representing 30% of non-muscle invasive bladder cancer cases, continues to be a challenging clinical problem. The five-year recurrence and progression rates of patients with the T1 disease are high at 42% and 20–40%, respectively [2]. Those with an increased depth of lamina propria invasion or with extensive lamina propria invasion are more than three times more likely to progress than patients with “superficial” invasion and have more than twice the risk of cancer-specific mortality [2, 3].

The treatment and decision-making processes are further complicated by several factors. For individuals staged with T1 or T2, there is a 40% risk of upstaging and a 5-year cancer specific survival rate of 88 and 63%, respectively [4, 5]. Further, the standard for high-risk T1 bladder cancer is radical surgery (cystectomy), and though these patients have a 80–90% cancer-specific survival at 5 years, 50–60% of patients have post-surgical complications, and their risk of mortality is 2–3% in the first 90 days after surgery [6–8]. Additionally, recovery is insubstantial with a known decline in health-related quality of life primarily due to the need for urinary diversion and the risks of sexual and bowel dysfunction [9]. In summary, patients are at risk for upstaging of non-muscle invasive bladder cancer, and thus are unnecessarily treated with radical surgery and live with its complications and quality of life decline. Consequently, clinicians and patients struggle with the choice of conservative bladder-preserving therapies versus radical cystectomy. Better tools are needed to risk stratify patients and provide more personalized treatment counseling.

We aim to develop an automated method to improve the staging accuracy and risk stratification of T1 bladder cancer. In [10, 11], a deep learning method was developed to automatically recognize muscularis propria, lamina propria, and urothelium from hematoxylin and eosin (H&E) stained bladder biopsies. It utilized a fine-tuned Inception v3 to classify patches of tissue into one of these three classes. Patch-wise predictions were aggregated into heatmaps and thresholded in order to achieve a segmentation of bladder layers. The method had good agreement with the pathologist but was limited due to several factors. First, the segmentation methodology was inefficient, as several hundred thousand overlapping tiles needed to be classified in order to produce a segmentation map for each slide. Second, the network often identified inflammation as urothelium, red blood cells as muscularis propria, and cautery artifacts for bladder layers. And third, the dataset was limited to only a few bladder biopsies, possibly lacking generalization. For these reasons, we sought a fully convolutional, multi-class semantic segmentation network on a larger dataset.

As a next step, we present a method to automatically identify anatomical structures from H&E-stained slides of bladder biopsies. Interpreting bladder anatomy from tissue biopsies is a crucial the first step for two reasons. First, clinicians need to identify anatomic structures in bladder biopsies that confirm T1 disease. Further, pathologists need to recognize the various landmarks within the lamina propria and the presence of tumor nuclei within the lamina propria in a precise manner to differentiate Ta (non-invasive papillary carcinoma) from T1 tumors. However, the prevailing literature suggests that pathologists struggle to consistently recognize lamina propria invasion from H&E bladder biopsies, often due to limitations such as anatomic variation, tissue size, fragmentation, and processing artifacts [12–16]. Thus, the ability to measure the depth of tumor invasion into bladder wall layers must be enhanced in order to reliably provide prognostic information that can guide treatment options. Here, as a first step towards enhancing the staging and risk stratification of T1 bladder cancer, we present an automated image analysis system to recognize major anatomical structures (urothelium, lamina propria, muscularis propria, and muscularis mucosa) from images of H&E-stained slides of bladder biopsies in addition to regions of red blood cells, cauterized, and inflamed tissues.

## Materials and methods

### Dataset

Our primary dataset consisted of 54 whole slide H&E images of T1 bladder biopsies, collected with the approval by the Ohio State University Institutional Review Board. All slides were anonymized and digitized at 40x magnification using a high-resolution scanner (Aperio ScanScope, Leica Biosystems) at 0.2437 μm per pixel. The dataset was randomly divided into two subsets – S1 (39 whole slide images) and S2 (15 whole slide images) datasets. Regions of each slide in S1 were annotated by an in-house pathologist, including urothelium (mucosa), lamina propria, muscularis propria, red blood cells (RBCs), cauterized tissue, inflammation, and muscularis mucosa. S2 was utilized as an independent test set; for this reason, none of the slides in S2 were annotated by the in-house pathologist.

**Fig. 1:**
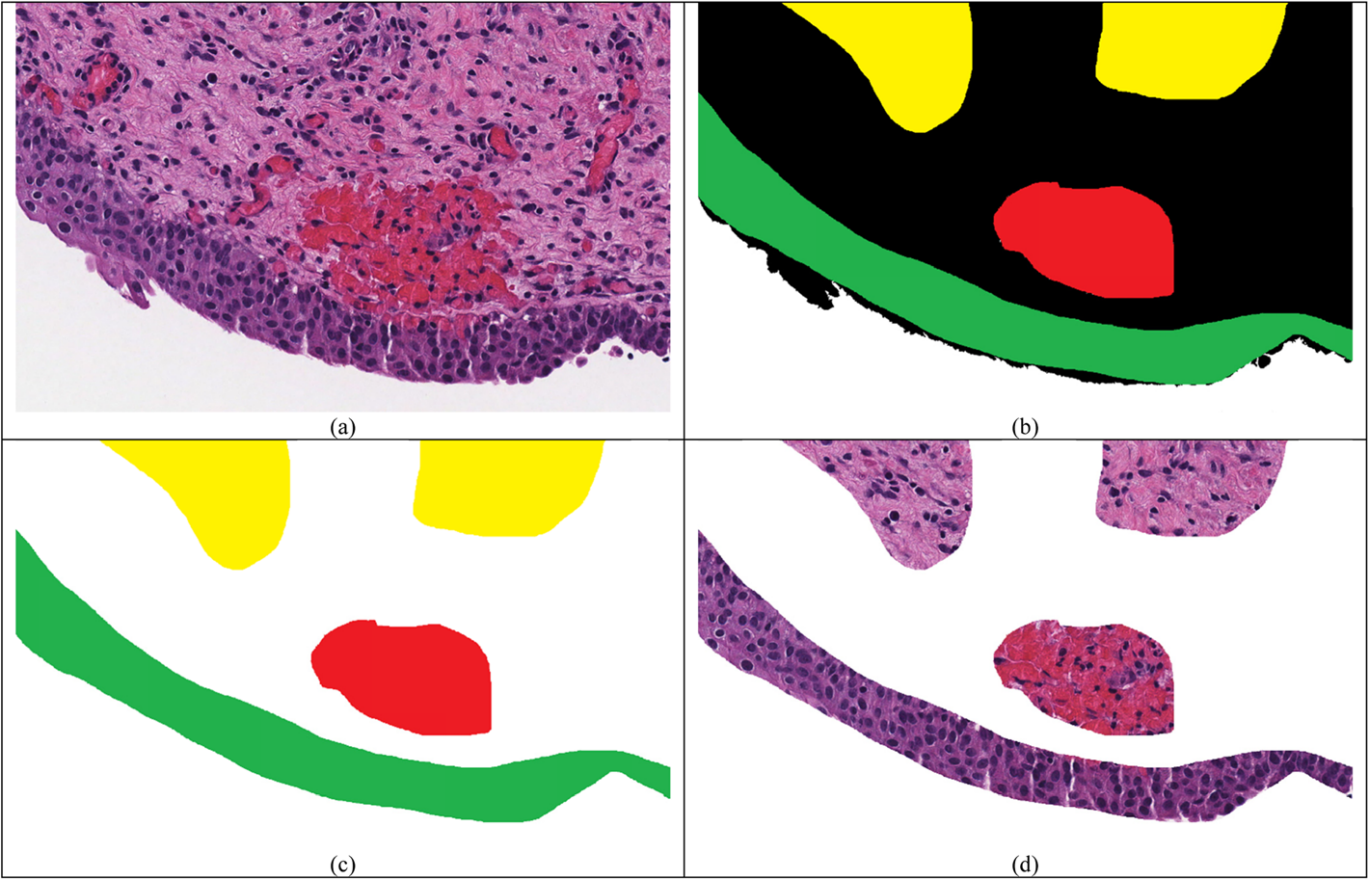
Ground truth preparation. **a**) Original image **b**) Ground truth prepared by the pathologist. Green, yellow, and red represent areas that were annotated by the pathologist as urothelium, lamina propria, and red blood cells. White corresponds to the unstained region while black correspond to the unlabeled region. **c**) We replaced the unlabeled region in (**b**) with background for computational ease. **d**) The image after post-processing that was used during training

While each slide in S1 was annotated, most tissue was unlabeled (unannotated) due to the tedious and time consuming nature of annotating a whole slide image. To prevent confusion during training, unlabeled regions of tissue were edited to look like background, i.e. they were removed from training. This way, the model only learned from labeled regions. Figure 1 visualizes background conversion of unlabeled regions.

S1 was then divided into training (31 slides), validation (4 slides), and testing (4 slides) sets randomly. Each slide was divided into 512 × 512 tiles with no overlap, paired with their annotations as the ground truth maps. In order to improve learning, tiles that contained more than 80% background, as well as tiles with no labelled data were labeled as background. As a result, S1 produced 13,335 pairs of images and ground truth masks. The number of tiles in each set was selected specifically to maintain an approximately 80:10:10 ratio between the training, validation, and testing sets, respectively.

Our secondary dataset consisted of 53 whole slide H&E images of T1 bladder biopsies. The image acquisition parameters for this dataset were the same as our primary dataset but were acquired at different point in time and thus serve as an additional independent testing dataset (similar to S2). None of the images in our secondary dataset were pre-annotated by the pathologist.

### U-net

U-Net is a convolution neural network (CNN) based semantic segmentation framework [17]. It consists of two parts. The first part, known as the contraction path, follows the architecture of a traditional CNN [16]. It consists of convolutional layers, each of which generates a certain number of filters, convolves them over an input image tile, and returns several feature maps. These feature maps are fed into a max pooling layer, which reduces the dimensionality of the feature maps by half. This process is repeated for each convolutional and pooling layer in the network. Overall, the contraction path serves to derive the context of an image.

The second part of U-Net is the expansion path. It consists of the same number of layers as the contraction path, but in place of a pooling layer, it uses an upscaling layer. Each layer takes the output of the previous layer, feeds it through a convolutional layer, and then upscales the result by a factor of two. This upscaled value is then concatenated with the feature map generated by the corresponding encoding layer prior to its pooling step. The final layer convolves a number of filters equivalent to the number of classes to classify, outputting a feature map containing a probability for each class. The result keeps track of the model’s confidence of classification for each pixel from the input.

### Modified U-net

**Fig. 2:**
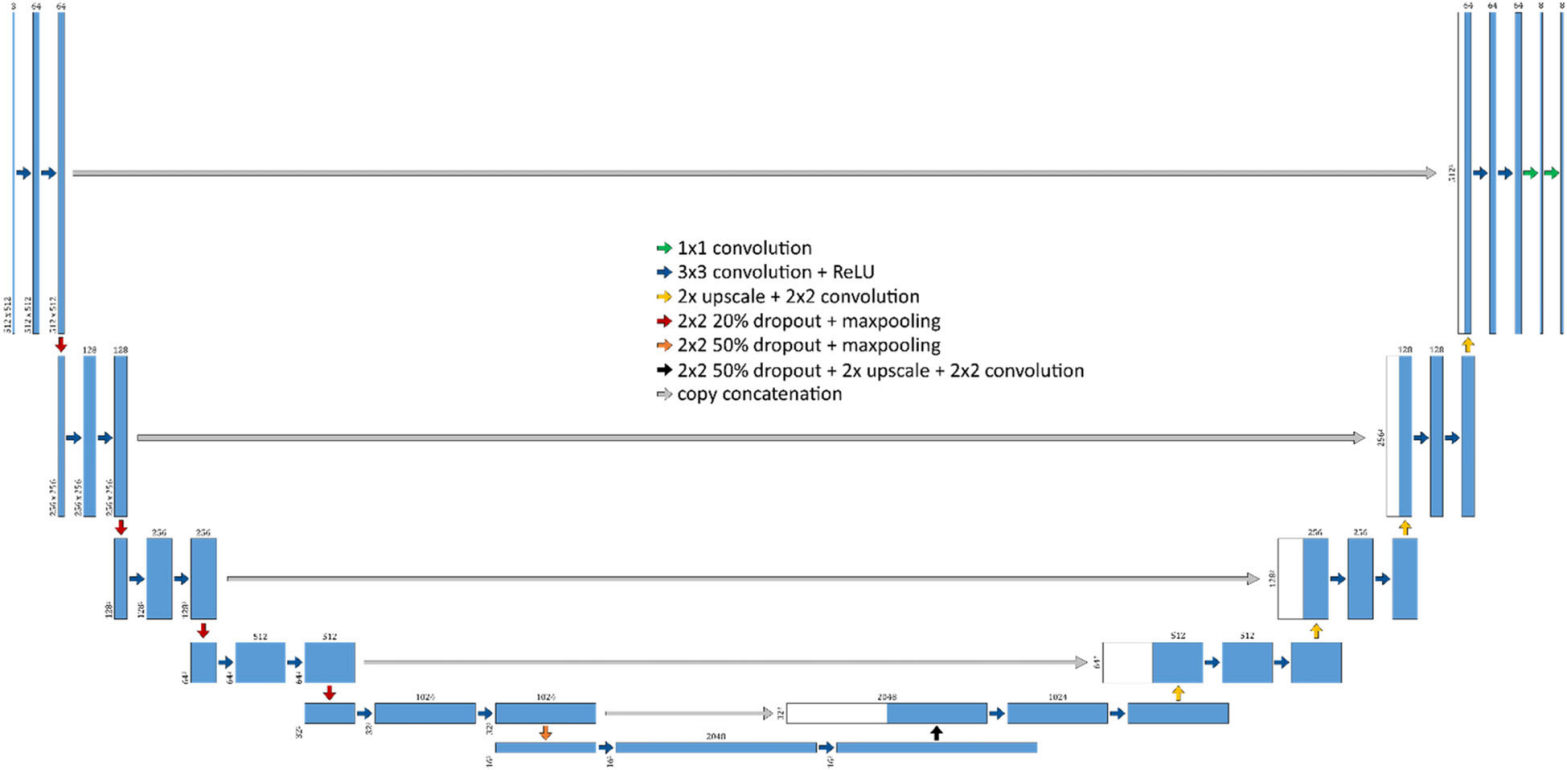
Modified U-Net architecture with 12 layers. Numbers on the top of blocks represent the number of feature maps, while numbers on the slide of blocks represent the feature map size

Our modified U-Net was implemented in Python and Keras with a Tensorflow backend. As Fig. 2 illustrates, its structure and organization are similar to the original U-Net implementation, encoding layers consist of two successive convolution and activation operations. Additionally, there are 64 filters in the first encoding, increasing by a power of two for each subsequent encoding layer. Unlike the original implementation, each encoding layer is first followed by random dropout to prevent overfitting. The first, second, a third encoding layers utilize a dropout of 20%, while the fourth and fifth encoding layers utilize 50%. The last five layers that make up the expansion path follow the original U-Net architecture. Categorical cross-entropy was utilized for loss due to the multi-class nature of our problem space, and Adam [18] was utilized as the optimizer. A model with eight, ten, and twelve layers were trained, tested, and validated with varying initializers (see Experimental Setup). These different configurations were tested in order to determine an appropriate receptive field (i.e. how much the context each set of feature maps see).

### Experimental setup

One important factor that determines the effectiveness of a deep learning model is how the layers are initialized [19]. An initializer defines how the initial random weights of a convolutional layer are set. Four different initializers were tested: *He Normal, He Uniform, Glorot Normal, and Glorot Uniform* [20]. Each method is similar but differ in two ways. The *He Normal* initializers samples a truncated normal distribution centered on 0, while the *Uniform* initializers use a uniform distribution within the bounds [−limit, limit]. The equation for the standard deviation of the normal distribution for the He initializer is $\sqrt{\frac{2}{Number\ of\ input\ units}}$, while for *Glorot* it is $\sqrt{\frac{2}{Number\ of\ \left( input\ units+ output\ units\right)}}$. The limits for *He normal/uniform* and *Glorot normal/uniform* are determined similarly, with $\sqrt{\frac{6}{Number\ of\ input\ units}}$ and $\sqrt{\frac{6}{Number\ of\ \left( input\ units+ output\ units\right)}}$, respectively.

The two experimental factors influencing the performance of our model were the initializers used in its convolutional layers as well as the total number of layers in the model. To select an appropriate model for our data, we tested four initializers (He Normal, He Uniform, Glorot Normal, Glorot Uniform) and three U-Net architectures comprising of eight, ten and twelve layers along with different amount of dropout.

An initializer that performs well with a U-Net with a fewer layers is not guaranteed to perform similarly in a U-Net with more layers. In order to determine the best combination of initializers and layers, each architecture was combined with each initializer and trained for 50 epochs on 31 training slides from S1. Generated predictions were compared pixel-by-pixel with ground truth masks to determine each model’s accuracy for each class. The best performing models were then tested on the validation and test dataset on S1. Moreover, the best performing models were used to automatically annotate 15 whole slide images in S2 dataset. These images were visually evaluated on a scale of 1–10 with an increment of 1 by a senior pathologist to determine our model’s accuracy in a clinical setting.

**Fig. 3:**
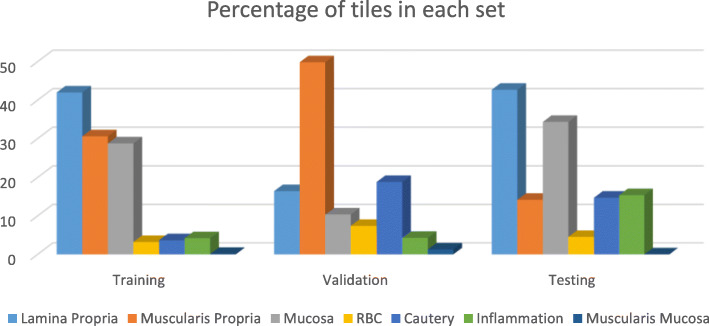
Percentage in tiles in each set

Table 1 shows the distribution of dataset S1 in each of the seven classes during training (Table 1a), validation (Table 1b), and testing (Table 1c). While there are relatively high number of tiles to properly train, validate and test lamina propria, muscularis propria and mucosa layers, there are only a limited number of RBC, cautery, inflammation, and muscularis mucosa tiles versus other categories. The differences in percentages of tiles in training, validation and test are due to different composition of the selected slides. Figure 3 depicts the relative percentage of each tile in each set.

**Table 1:** The number of tiles in the training, validation, and test slides. Each tile is of size 512 × 512 pixels

	Lamina Propria	Muscularis Propria	Mucosa	RBC	Cautery	Inflammation	Muscularis Mucosa
**Training set** **(31 slides)**	5076	3702	3474	389	444	507	14
**Validation set** **(4 slides)**	206	625	130	93	236	54	16
**Testing set** **(4 slides)**	461	153	371	49	159	166	0

## Results and discussion

Due to space constraints, only the result of the best performing models is reported, i.e., He Normal with eight and twelve layers. An additional spreadsheet with comprehensive results for each initializer and U-Net configuration combination can be found in [Additional file 1]. Table 2 reports the results of using eight layered architecture initialized with He Normal on the validation and test sets in S1. It is clear that the model performed well in identifying the three bladder layers and background. However, it struggled to correctly classify pixels in cauterized and inflamed tissue. We attribute this deficit to the observation that these classes contained the smallest number of training samples. Although it seems the model did well to classify muscularis mucosa, there were few training and validation/testing samples. Thus, this needs to be validated on a dataset with large amount of muscularis mucosa.

**Table 2:** Results (pixel level accuracy) on 8 layered U-Net architecture initialized using He Normal. a) Results on the validation slides in dataset S1. b) Results on the test slides in dataset S1

	a			b		
	Accuracy	True Positive	False Negative	Accuracy	True Positive	False Negative
**Background**	0.99	94,424,339	54,224	0.99	119,213,206	48,864
**Lamina Propria**	0.99	28,939,686	177,916	0.97	52,727,211	1,696,663
**Muscularis Propria**	0.88	110,788,573	14,326,899	0.90	22,782,045	2,649,277
**Mucosa**	0.87	11,433,732	1,750,370	0.90	37,076,397	4,034,633
**RBC**	0.92	9,996,911	848,300	0.93	1,884,697	146,841
**Cautery**	0.62	6,424,900	3,927,745	0.28	6,701,447	16,835,934
**Inflammation**	0.94	41,268,732	2,686,066	0.52	9,082,821	8,235,484
**Muscularis Mucosa**	0.89	1,511,039	169,144	N/A	N/A	N/A

Table 3 reports the results on validation and test sets from S1. As in the result presented in Table 2, the model worked decently in identifying the three bladder layers. However, it struggled to identify cauterized and inflamed tissue, again due to limited training samples in our dataset.

**Table 3:** Results (pixel level accuracy) on 12 layered U-Net architecture initialized using He Normal. a) Results on the validation slides in dataset S1. b) Results on the test slides in dataset S1

	a			b		
	Accuracy	True Positive	False Negative	Accuracy	True Positive	False Negative
**Background**	0.99	91,488,149	672,063	0.99	119,216,562	45,508
**Lamina Propria**	0.99	27,861,365	278,336	0.98	53,285,338	1,138,536
**Muscularis Propria**	0.87	105,691,097	15,302,863	0.88	22,362,961	3,068,361
**Mucosa**	0.90	11,391,120	1,289,356	0.97	39,830,580	1,280,450
**RBC**	0.99	10,701,972	143,239	0.93	1,896,309	135,229
**Cautery**	0.64	6,612,587	3,740,058	0.41	9,610,122	13,927,259
**Inflammation**	0.97	41,619,905	1,393,986	0.41	7,082,849	10,235,456
**Muscularis Mucosa**	0.84	1,371,094	258,490	N/A	N/A	N/A

Overall, key bladder layers (mucosa, lamina propria, and muscularis propria) are identified with high accuracy across several experimental setups. These numerical results provide substantial evidence that the proposed methodology performs well for accurate multi-class segmentation. Further, the configurations reported in Tables 2 and 3 performed considerably better than other configurations [Additional file 1], providing quantitative evidence for the eminence of these particular configurations.

**Fig. 4:**
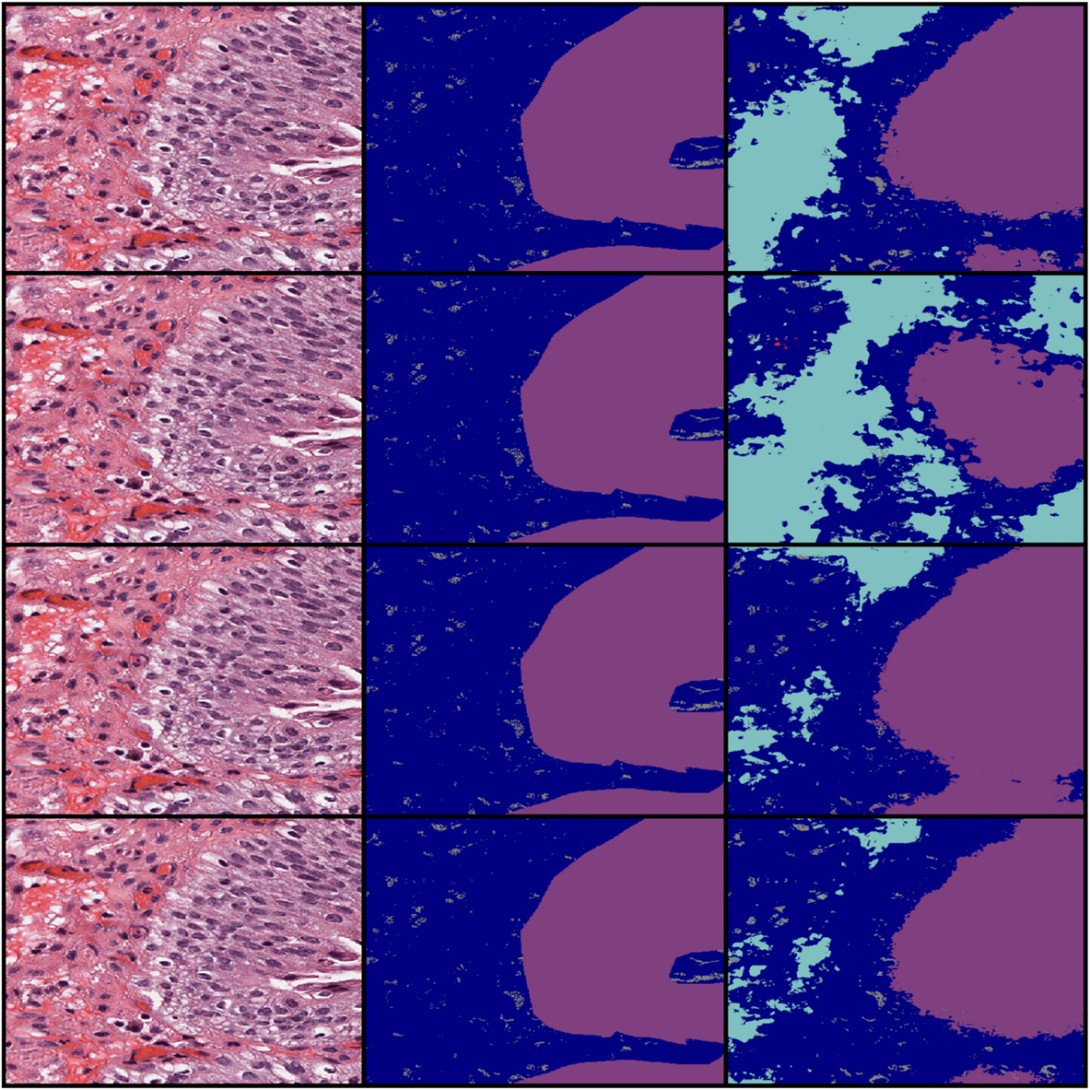
Prediction Results. Leftmost column is the input image, middle column is the ground truth, and rightmost is the prediction. The rows in descending order are: Glorot Normal, Glorot Uniform, He Normal, and He Uniform*.* The color purple is mucosa, light blue is lamina propria, dark blue is unlabeled, and gray is background

Figure 4 illustrates a sample of the predictions made by the models trained on each initializer using a 12 layer U-net. The trend that can be seen here, and that is also reflected across all predictions made by these models, is that the Glorot initialized models tend to be more liberal with the boundaries of bladder regions, while He initialized models are more conservative. Qualitatively, He Normal seemed to be the best initializer. Based on these results, we determined that He Normal was the best initializer. A 7-fold cross validation was performed using a 12 layer U-net architecture to test the generalizability of our modified U-net model (Table 4).

**Table 4:** 7-fold cross validation of dataset S1 using He Normal initializer and a 12 layer U-net architecture. Expressed a mean accuracy and standard deviation (std)

Background	Lamina Propria	Muscularis Propria	Mucosa	RBC	Cautery	Inflammation	Muscularis Mucosa
99.95 ± 0.03	97.67 ± 0.72	97.53 ± 1.4	93.57 ± 2.7	84.61 ± 12.92	55.09 ± 9.94	75.69 ± 17.84	N/A

The 15 whole slide images in S2 were segmented with our best performing model and visually evaluated by an experienced pathologist on a scale of 1 to 10 with a step of 1. Each slide in S2 contained from 1 to 3 tissue sections. The pathologist gave a score of 8.9 ± 0.6 for segmentation accuracy. Furthermore, it only took 23 min for the pathologist to assess 15 slides. This is far less time than would elapse if a pathologist were to prepare a ground truth from scratch.

As the segmentation results on S2 were evaluated by the pathologist who originally annotated the images in S1, there is the possibility of an element of “evaluation bias.” To circumvent this issue and to support the generalization of our proposed model to pathologists with difference in training and experience, we asked a pathologist from another institution to evaluate our segmentation results on the secondary dataset. All of the images in the secondary dataset were pre-annotated by the method. The pathologist gave a score of 8.9 ± 0.6 for segmentation accuracy. This shows that the segmentation results of the proposed method are acceptable across different institutions. Additionally, the narrow range of error indicates that there is little clinical variance in our segmentation results. However, as the pathologists were reviewing slides that were already annotated by the method, there still exists an element of bias (i.e. the model biases the pathologists).

The pathologist noted that in some instances, the proposed method failed to recognize grandular cells within epithelium. They also noted that the method struggled to identify sclerotic stroma and vessels present within smooth muscles. In lamina propria, the method struggled whenever it encountered necrotic regions. Unfortunately, only a few images in the training dataset contain examples of these misclassified regions. However, we expect that more thorough annotations on a relatively larger dataset with help us overcome these limitations.

## Conclusions

We present a modified U-Net based multi-class segmentation method to identify different anatomical regions in T1 bladder cancer biopsies. From a technical point of view, we modified the U-Net base model by adding some additional convolutional layers to the network. We also introduced dropout modules after certain layers in the network to minimize the effect of overfitting. We also compared the effect of using different initializers on a multi-class segmentation problem. The method’s ability to accurately segment bladder layers has the potential to minimize the time needed by pathologists to review the bladder slides.

From the results, we can conclude that the Glorot initialized models tend to be more liberal with the bounds of their regions, while He initialized models are more conservative. On an independent set of 15 slides (dataset S2) that were pre-annotated by our model, it only took 23 min for the pathologist to review 15 slides. Moreover, the algorithm has the potential to identify the bladder layers accurately and hence can assist the pathologist with the diagnosis of T1 bladder cancer.

In the future, we intend to experiment with other semantic segmentation networks as well as acquire a larger set of images. For a problem as complex as our, U-net (and other semantic segmentation networks) require more examples of different tissue structures in order to be able to generalize to unseen data. Once meaningful segmentation between bladder layers is achieved, we will develop a method to localize tumor cells in lamina propria, automatically measure their invasiveness into lamina propria, and correlate invasion with clinical outcome to ultimately substage T1 bladder cancer to improve clinical decisions.

## Supplementary information

**Additional file 1.**

## Data Availability

The datasets generated during the current study are not publicly available due to sheer file size but are available from the corresponding author on reasonable request. Code is available at https://github.com/cialab/Bladder_U-net .

## Abbreviations

H&E: Hematoxylin and eosin
RBC: Red blood cell
CNN: Convolutional neural network
